# Screening of Metabolites and Metabolic Pathways in Five Different *Ocimum* Species From the Same Origin Using GC-MS

**DOI:** 10.1155/bri/7121687

**Published:** 2025-06-24

**Authors:** Ravi Prakash Jaiswal, Vishal Chugh, Sushil Nagar, Shalini Purwar, Akbare Azam, Ankit Verma

**Affiliations:** ^1^Department of Chemistry, Government Girl's P.G. College, Ghazipur 233001, Uttar Pradesh, India; ^2^Department of Basic and Social Sciences, College of Horticulture, Banda University of Agriculture and Technology, Banda 210001, Uttar Pradesh, India; ^3^Department of Biochemistry, College of Basic Sciences and Humanities, CCS Haryana Agricultural University, Hisar 125004, Haryana, India; ^4^Department of Basic and Social Sciences, College of Forestry, Banda University of Agriculture and Technology, Banda 210001, Uttar Pradesh, India

**Keywords:** caryophyllene, estragole, eugenol, GC-MS, linalool, *Ocimum* species

## Abstract

This study presents the comparative and preliminary phytochemical analysis of essential oils extracted from 5 different *Ocimum* species, including *Ocimum basilicum* Linn, *Ocimum canum* Sims, *Ocimum citriodorum*, *Ocimum gratissimum* Linn *and Ocimum sanctum* Linn. The gas chromatography coupled with single quadrupole mass spectrometry was employed for the screening of the different metabolites. The present study investigates a total number of 111 bioactive compounds which were identified across the five *Ocimum* species, with *O. citriodorum* exhibiting the highest diversity. The analysis revealed significant variations in the chemical profiles, attributed to differing eco-climatic conditions. Key bioactive compounds, such as *α*-pinene, linalool and caryophyllene, were consistently found across species. The study also mapped these compounds to metabolic pathways, highlighting their roles in monoterpenoid, phenylpropanoid and sesquiterpenoid biosynthesis. Detailed analysis of *O. basilicum*, *O. citriodorum*, *O. sanctum*, *O. canum* and *O. gratissimum* oils demonstrated unique metabolic properties, with each species showing distinct pathway activations and dominant compounds. The findings were validated using principal component analysis and hierarchical clustering analysis, confirming the species' chemical diversity and distinct clustering. This comprehensive characterization enhances our understanding of *Ocimum* spp. essential oils, offering valuable insights for their pharmaceutical, food and cosmetic applications.

## 1. Introduction

The genus *Ocimum*, family Lamiaceae, is a well-known aromatic and rich essential oil-bearing plant family. More than 111 species are grown widely and distributed throughout tropical and temperate regions and are collectively known as the ‘basils', Tulsi (Urdu language). From ancient times to modern research, this plant has made significant contributions to the field of science. It contains various bioactive compounds, including aromatic esters, phenolics, terpenes and alkaloids, which have led to numerous medicinal applications. These applications include insecticidal, antimicrobial, repellent, larvicidal, nematocidal, antioxidant and therapeutic uses. Therapeutic benefits encompass anti-inflammatory, antinociceptive, antipyretic, antiulcer, cardioprotective, anthelmintic, immunomodulatory, analgesic, anticarcinogenic, skin permeation enhancement and antilipidemic properties [1–5]. *Ocimum gratissimum*, *Ocimum basilicum* and *Ocimum sanctum* are commonly known as clove basil (wild basil/East India basil), holy basil and sweet basil, respectively. The several countries of East Asia, Europe, America and Australia are frequently cultivating these *Ocimum* species for the production of essential oils. Another species *Ocimum americanum* (*O. americanum*), formerly known as *Ocimum canum* (*O. canum*), includes a wild species in India, and its essential oil is used for several profitable purposes [6, 7]. These species have varied medicinal properties due to their high biological activities, which are used in various traditional and native medicines. In addition to these, they are also apparent for their distinctive composition of flavour/aroma due to their high-quality essential oils and aroma chemicals appreciated in food, cosmetic, fragrance and pharmaceutical industries [8–11]. The essential oil composition of *Ocimum* taxa is very complex and shows wide-ranging composition variations due to different morphotypes, genotypes, chemotypes, environmental/climatic conditions and varied agronomical factors with cultivars within the taxa [12].

Chemical characterization can separate accelerations based on the concentration of a particular substance and determine the inherent variability or variability between subscriptions of the same species. Sometimes, *O. gratissimum* and other chemotypes of *O. tenuiflorum* [6, 13] and tarragon-rich (87%) were reported as chemotypes of *O. tenuiflorum* in Australia [14]. The chemical composition of essential oils in the genus *Ocimum*, particularly the plant of *O. basilicum*, has been the subject of many studies because chemotaxis can also be used to assess interspecies or interspecies variability [15]. The composition of the ether oils of *Ocimum* gave rise to a comprehensive variety of oil components, and various chemical types have been reported from different regions of the world. The major components distributed in *Ocimum* species are phenylpropanoids (eugenol, methyleugenol, tarragon, methylcinnamic acid), monoterpenoids (linalool, 1,8-cineole, camphor, thymol) sesquiterpenoids mainly *β*-caryophyllene, germacrene D, *β*-selinene, *β*-bisabolene, *α*-selinene and elemol [16–19].

The aim of this study was to chemically quantify ether oils of *Ocimum* species for the examination of major molecules such as estragole, caryophyllic, linalol and eugenol of five *Ocimum* species, namely, *Ocimum* species, viz. *Ocimum basilicum* Linn*, Ocimum citriodorum*, *Ocimum canum* Sims, *Ocimum gratissimum* Linn and *Ocimum sanctum* Linn. This study first used gas chromatography–mass spectrometry (GC-MS) analysis to evaluate essential oils of *Ocimum* species to quantify and identify target molecules, and show all *Ocimum* species that do not characterize and identify molecules at the same time, but exhibit detailed variation in the composition. Gas chromatography combined with a single quadrupole mass spectrometry (GC-MS) and identification of their metabolic pathway by Kyoto Encyclopedia of Genes and Genomes (KEGG).

## 2. Materials and Methods

### 2.1. Sampling and Isolation of Essential Oils

Five different species of *Ocimum* seeds like *O. basilicum*, *O. canum*, *O. sanctum*, *O. citriodorum* and *O. gratissimum* were collected from the National Seed Bank of NBPGR, New Delhi. Plants were grown in an open area of 100 × 2500 cm (plot size) with plant spacing of 50 cm^2^ located at Government Girls P.G. College, Ghazipur. The plot was drip irrigated for 2 h, 3 times per week with a dose of fertilizing (Urea [46-0-0] and NPK fertilizer [15-15-15] in the ratio 1:3) once a month until flowering. These essential oils, consisting of fresh herbs, were extracted from the *Ocimum* species' inflorescence (flower) parts. The extraction process was carried out through hydrodistillation using a Clevenger apparatus for a duration of 3 h. The extracted essential oils were quantified directly within the extraction burette. Subsequently, the oil samples underwent dehydration using anhydrous sulphate and were stored at low temperatures in the fridge for further analysis [20].

### 2.2. Sample Preparation

Before quantifying the molecules in essential oils of *Ocimum* species, approximately 0.2 g of each sample was meticulously measured and placed into 20-mL amber-coloured volumetric flasks. To ensure proper mixing, 5 mL of methanol (specifically LC-MS CHROMASOLV, ≥ 99.9% purity, obtained from Merck) was added and homogenized. The solution was then brought to the desired volume. Subsequently, the solution was passed through a 0.45-μm membrane filter, and 0.5 mL of the filtrate was employed as the testing solution for screening the molecules present in basil oil samples using GC-MS [21].

### 2.3. GC-MS Qualitative Analysis

The GC-MS was performed, as per the method already streamline by Jaiswal et al. [22]. Briefly, the methanolic extract analysis of oil samples isolated from different *Ocimum* species through GC-MS was conducted using an Agilent 8890 gas chromatograph coupled with a model 5977B GC/MSD (gas chromatograph/mass spectrometry detector), featuring an HP-5 MS capillary column having length of 30 m, with an inner diameter of 250 μm and a film thickness of 0.25 μm. Helium gas was used as the carrier gas, with a flowing rate of 1 mL/min and a split ratio of 1:100. The temperature profile of the oven was programmed to increase from 60°C to 240°C at a rate of 3°C per minute. Subsequently, it was raised to 270°C at a rate of 5°C per minute, followed by a postrun period of 4 min at 305°C. The injector and mass transfer line temperatures were held at 250°C [23].

In the MS analysis, electron impact ionization mode (EI) at 70 electron volts (eV) was utilized, with a mass scan range spanning from 30 to 500 *m*/*z* (mass-to-charge ratio) at a sampling rate of 1.0 scan per second. The mass source and quadrupole (Quad) temperatures were set at 230°C and 150°C, respectively. For the identification of volatile compounds within the basil essential oil, data processing involved comparing the retention indices (RI), which were determined using a C_7_–C_30_ Saturated Alkanes Calibration Standard from Sigma-Aldrich, St. Louis, MO, USA, with the mass spectra fragmentation patterns of each compound. These were matched with the RI and mass spectra information in the Wiley Registry 12th Edition/NIST 2020 Mass Spectral Library. This analysis used the Mass Hunter Workstation Qualitative Analysis Version 10.0 software developed by Agilent Technologies, Palo Alto, CA, USA (22).

### 2.4. Qualitative Analyses and Statistical Evaluation of Analytical Data

The results were subjected to statistical evaluation using Agilent Mass Hunter WorkStation Mass Profiler Professional (MPP) Version 15.1.

### 2.5. Metabolic Pathway Analysis by KEGG

The sample pathway analysis was carried out using the pathway analysis function available at the KEGG web server. This function combines enrichment and topology analysis to estimate the possible biological influences based on the perturbed pathways.

## 3. Results

### 3.1. Identification of Basil Oil Compounds in *Ocimum* Species

Remarkably, there has been no previous report on plant metabolic characterization using GC-MS to unveil the presence of various bioactive compounds in extracts of different basil species. Thus, this study was conducted to fill this knowledge gap. During the GC-MS analysis, 111 distinct peaks were observed in the essential oils of various basil species. Each of these peaks represented bioactive compounds, and their identities were established by correlating their retention times and molecular formulas with known compounds as proposed by the NIST library (Table 1). Notably, *O. citriodorum* exhibited the highest number of compounds (51 compounds) in its extract, followed by *O. basilicum* (36 compounds), *O. sanctum* (28 compounds) and *O. gratissimum* (20 compounds) (Table 1). These variations in chemical composition among different basil species may be attributed to their origin in diverse climatic and soil conditions.

Our findings highlight the biological significance of most of the identified compounds. The identification and separation of constituents in basil oils from 5 distinct *Ocimum* spp. were achieved using the GC-MS protocol developed for this purpose. This was based on the retention index determined with a series of *n*-alkanes (C_7_–C_30_) calibration standards. The analysis was conducted under identical experimental conditions, involving coinjection with standards or known essential oil constituents, a mass spectra library search, and comparing the mass spectral and retention data with the existing literature. The relative quantities of distinct components were determined using the peak area normalization and expressed in percentages (%). This comprehensive analysis sheds light on the diverse and biologically important compounds in the essential oils of different basil varieties, making it a valuable resource for multiple industries.

### 3.2. Analysis of Metabolic Pathways of Different *Ocimum* Varieties

**Figure 1:**
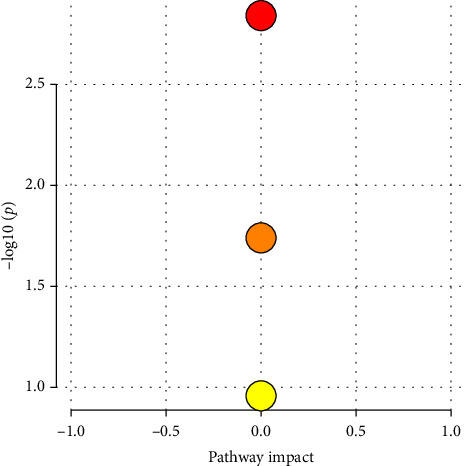
All five *Ocimum* species collectively exhibit several key metabolic pathways. The phenylpropanoid pathway, marked in yellow, plays a vital role. The monoterpenoid pathway, highlighted in orange, is another significant feature, contributing to the diverse biochemistry of these plants. Additionally, the sesquiterpenoid and triterpenoid biosynthesis pathways, represented by the red colour, are actively present across these *Ocimum* species.

**Figure 2:**
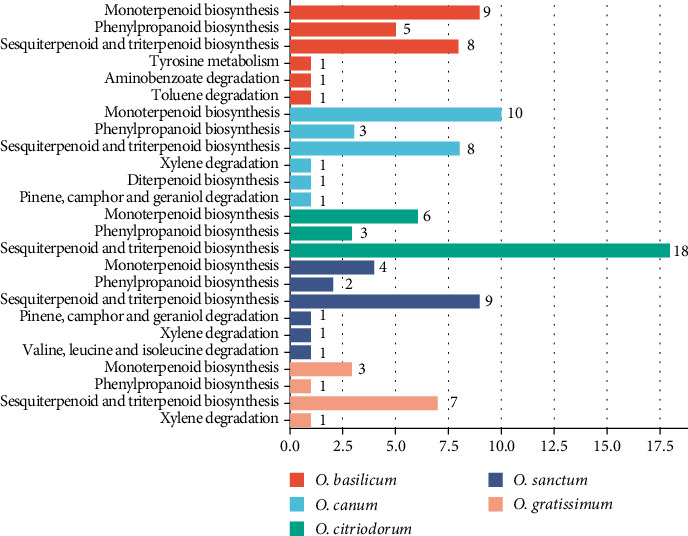
KEGG analysis of metabolic pathways present in five different *Ocimum* species.

Table 1 provides a comparative list of compounds found across different species. The chemical analysis of *Ocimum* essential oils revealed the presence of 110 volatile constituents in all five basil varieties. Compared with the KEGG web server database, it was found that the specific metabolites in the experiment were primarily involved in monoterpenoid biosynthesis, phenylpropanoid biosynthesis, sesquiterpenoid and triterpenoid biosynthesis (Figures 1 and 2). Based on our analyses, some compounds were consistently found in significant quantities among the different species, including *α*-pinene, *α*-elemene, *α*-neoclovene, D-limonene, (E)-linalool oxide A, cis-linalool oxide, *δ*-cadinene, *α*-cubebene, linalool, eugenol, methyleugenol, estragole, caryophyllene, aromadendrene, *α*-humulene, *α*-bisabolene, acetyl eugenol, caryophyllene oxide and caryophyllenyl alcohol. The specific metabolites dedicatedly present in specific species of the *Ocimum* variety are shown in Supporting Tables S1–S5.

### 3.3. Analysis of Metabolic Pathways and Properties of *O. basilicum*

**Figure 3:**
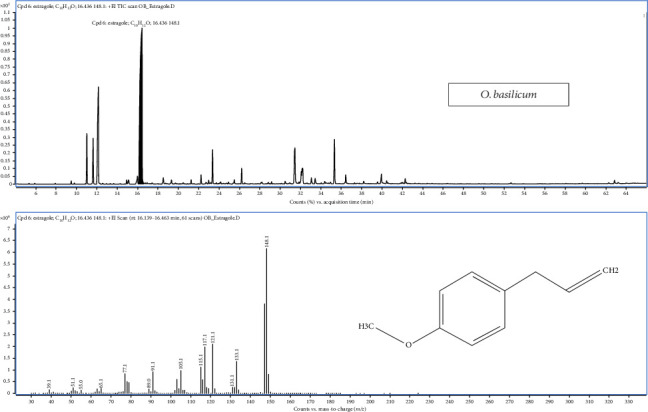
GC-MS total ion chromatograms (TIC) of essential oil of *Ocimum basilicum* Linn.

The chemical composition of *O. basilicum* oil was found to comprise a total of 47 compounds, and the chromatogram confirms it as estragole-rich, with a major peak at 16.46 min (Figure 3). The specific metabolites of *O. basilicum* were determined in six specific pathways, like monoterpenoid biosynthesis, phenylpropanoid biosynthesis, sesquiterpenoid and triterpenoid biosynthesis, aminobenzoate degradation, tyrosine metabolism and toluene degradation (Figure 2). The chromatogram showed that the predominant compounds in this basil oil were estragole, the dominant constituent in phenylpropanoid biosynthesis, accounting for a substantial percentage of the oil (49.49%–61.05%). Additionally, the other major compounds included (E)-linalool oxide A (ranging from 0.95% to 3.73%), linalool (14.3%–20.33%), *α*-longipinene (1.26%–1.83%) and 4-methoxycinnamaldehyde (2.77%–5.19%). Among these pathways, aminobenzoate degradation, tyrosine metabolism and toluene degradation only occur in *O. basilicum*.

### 3.4. Analysis of Metabolic Pathways and Properties of *O. canum*

**Figure 4:**
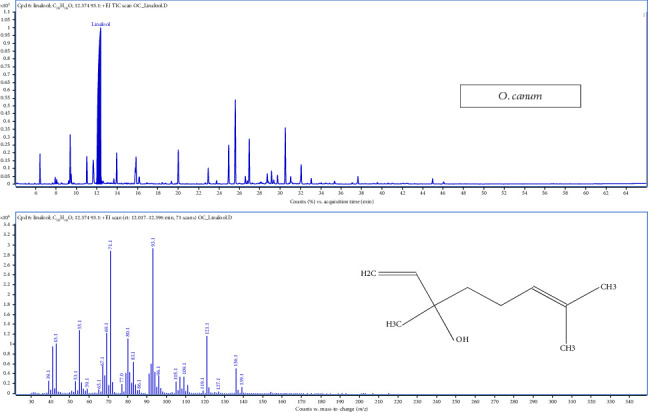
GC-MS total ion chromatograms (TIC) of essential oil of *Ocimum canum* Linn.

The chromatogram of GC-MS analysis of *Ocimum canum* (Figure 4) identified 39 compounds, confirming it as a linalool-rich sample with a major peak at 12.37 min. KEGG analysis of compounds revealed that six pathways were activated in *O. canum* involved in monoterpenoid biosynthesis, phenylpropanoid biosynthesis, sesquiterpenoid and triterpenoid biosynthesis, xylene degradation, diterpenoid biosynthesis and pinene, camphor and geraniol degradation (Figure 2). Table 1 shows that monoterpene hydrocarbons were the dominant compounds, with linalool accounting for the largest proportion (ranging from 50.81% to 51.26%). Caryophyllene (7.66%–7.98%) and *α*-humulene (3.71%–3.8%) were identified as major sesquiterpene compounds in *O. canum* oil. Other compounds present in noteworthy quantities included *α*-pinene (1.59%–1.61%), D-limonene (3.05%–3.3%), (E)-linalool oxide A (1.94%–2.06%), eugenol (1.15%–1.39%), methyleugenol (2.92%–2.94%) and *α*-bisabolene (4.53%–5.22%).

### 3.5. Analysis of Metabolic Pathways and Properties of *O. citriodorum*

**Figure 5:**
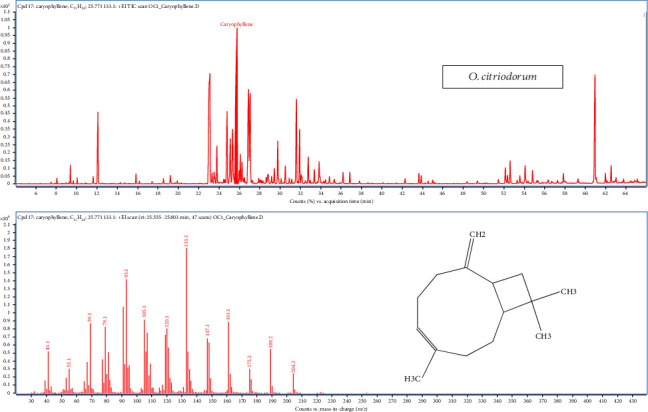
GC-MS total ion chromatograms (TIC) of essential oil of *Ocimum citriodorum*.

The results presented in Table 1 highlight that *O. citriodorum* essential oil was particularly rich in volatile compounds, with a total of 53 different compounds identified and a chromatogram, where a prominent peak at approximately 25.77 min corresponds to caryophyllene, identified based on its retention time and intensity (Figure 5). These are related to three pathways: monoterpenoid biosynthesis, phenylpropanoid biosynthesis, sesquiterpenoid and triterpenoid biosynthesis (Figure 2). Caryophyllene was the major sesquiterpene compound, constituting 17.87%–17.93% of the oil. Other compounds found in significant amounts included *α*-neoclovene (3.52%–3.79%), *γ*-cadinene (1.23%–1.25%), D-limonene (0.64%–2.64%), aromadendrene (7.11%–7.17%), *α*-humulene (6.02%–6.1%), linalool (3.62%–5.62%), eugenol (11.5%–13.5%), copaene (1.59%–1.67%), cinnamaldehyde dimethyl acetal (2.38%–4.17%), *α*-elemene (2.55%–2.75%), *α*-longipinene (1.06%–1.07%), *δ*-cadinene (1.99%–2.02%), fonenol (2.37%–2.61%), caryophyllenyl alcohol (4.78%–4.83%), ledol (1.31%–1.36%), acetyl eugenol (1.06%–1.08%) and rosifoliol (6.68%–6.87%).

### 3.6. Analysis of Metabolic Pathways and Properties of *O. sanctum*

**Figure 6:**
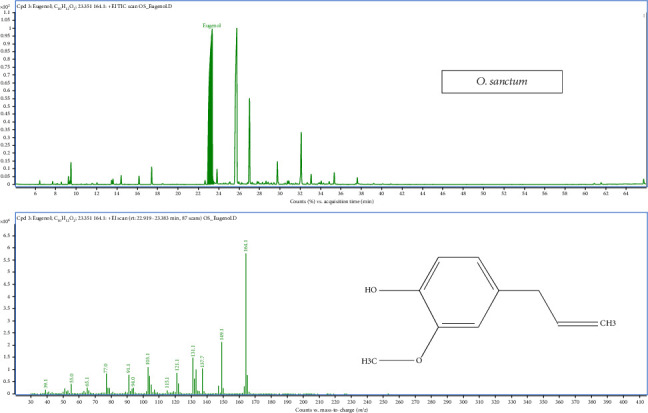
GC-MS total ion chromatograms (TIC) of essential oil of *Ocimum sanctum* Linn.

In a similar vein, the analysis of *O. sanctum* essential oil identified 31 volatile compounds and six metabolic pathways including monoterpenoid biosynthesis; phenylpropanoid biosynthesis; sesquiterpenoid and triterpenoid biosynthesis; pinene, camphor and geraniol degradation; valine, leucine and isoleucine degradation; and xylene degradation that constituted the entire oil composition, as depicted in Figures 2 and 6. Sesquiterpenoid and triterpenoid biosynthesis has a major pathway with nine compounds present. The major sesquiterpene compound in *O. sanctum* oil was caryophyllene (25.43%–26.16%). Other compounds present in noteworthy amounts included eucalyptol (1.01%–1.05%), citronellol (0.88%–0.97%), copaene (0.89%–0.93%), *α*-humulene (6.91%–6.97%) and *δ*-cadinene (1.43%–1.5%).

### 3.7. Analysis of Metabolic Pathways and Properties of *O. gratissimum*

**Figure 7:**
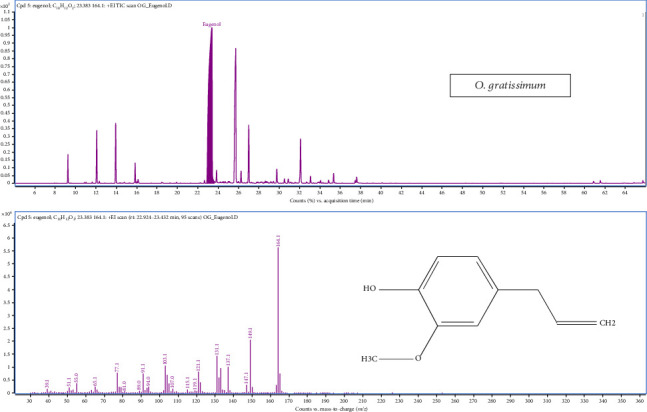
GC-MS total ion chromatograms (TIC) of essential oil of *Ocimum gratissimum* Linn.

The analysis of O. gratissimum essential oil identified 23 compounds, with the chromatogram showing a dominant peak at 23.35 min corresponding to eugenol, confirmed by its retention time and peak intensity (Figure 7), and four metabolic pathways such as monoterpenoid biosynthesis, phenylpropanoid biosynthesis, sesquiterpenoid and triterpenoid biosynthesis and xylene degradation (Figure 2). In this case, phenylpropanoids also dominated the oil composition, with eugenol being the major constituent, constituting a significant proportion (58.67%–59.83%). Additionally, the major sesquiterpene compound identified was caryophyllene (ranging from 17.69% to 18.61%), while monoterpene hydrocarbons such as linalool were present in the range of 3.38%–3.33%. Other compounds found in notable quantities included p-cymene (1.42%–1.57%), 2-bornanone (3.87%–3.89%), *α*-terpineol (ranging from 1.18% to 1.19%), *α*-humulene (4.25%–4.41%) and caryophyllene oxide (ranging from 2.38% to 3.36%).

### 3.8. Exploring the Chemical Diversity of Different *Ocimum* spp.

**Figure 8:**
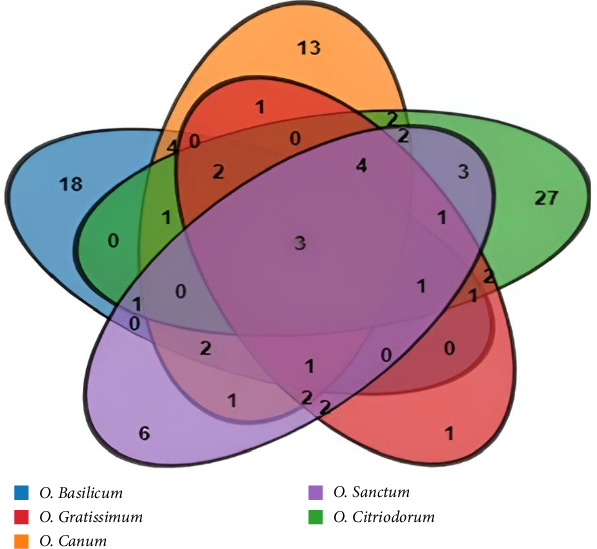
Venn diagram illustrating the origin and distribution of compounds in five distinct *Ocimum* species within the same genus.

Table 1 and Supporting Tables S1–S5 show that different *Ocimum* species have specific metabolites. However, the selected five *Ocimum* species were of same origin, but they had their own properties, and their metabolic pathways and mechanisms were still different. The Venn analysis approach was used to investigate and compare the compound compositions of different *Ocimum* species. The intricate interplay of phytochemicals within these plants, illustrated in Figure 8, revealed the distinctive and shared compounds among the various species. This comparative examination significantly enriches our understanding of the chemical profiles inherent to *Ocimum*. Interestingly, when comparing all five species, only three compounds (α-humulene, caryophyllene and caryophyllene oxide) were shared, suggesting a common origin for these *Ocimum* varieties. This comparative analysis highlights that the chemical distinctions among these species, such as those observed in *O. basilicum*, *O. sanctum*, *O. canum*, *O. citriodorum* and *O. gratissimum*, underscore their unique properties and potential applications.

### 3.9. Principal Component Analysis (PCA) and Hierarchical Clustering Analysis (HCA)

**Figure 9:**
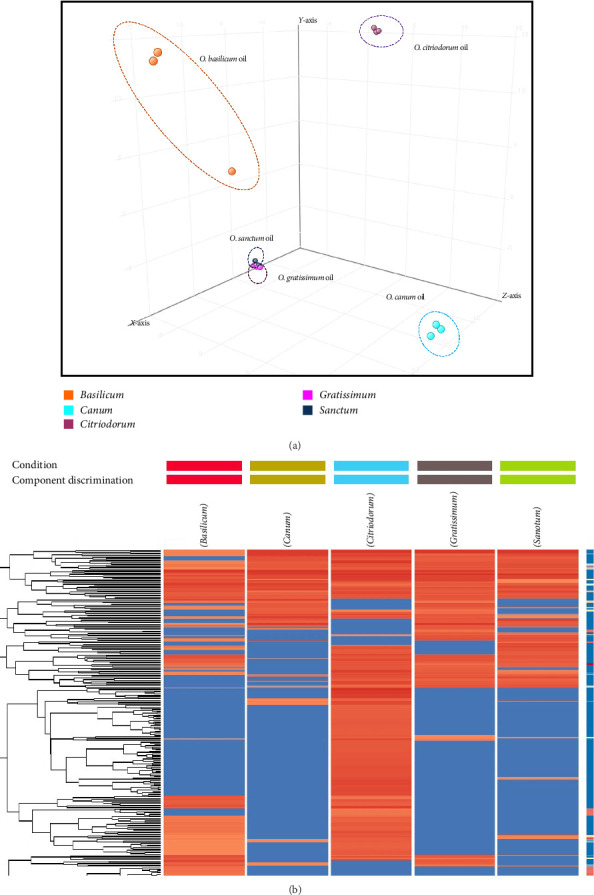
(a) 3D principal component analysis (PCA) of five varieties of basil essential oil. (b) Hierarchical clustering analysis (HCA) heat map for the association of compounds detected in five different varieties of basil oil.

PCA was used as an unsupervised statistical tool to reduce the dimensionality of large data sets to reveal differences between the volatile compositions and *Ocimum* essential oil varieties. A total of one hundred and ten volatile selected compounds were subjected to 3D PCA. The first three principal components account for approximately 80% of the total variance in the original data (36.4%, 27.57% and 15.53%, respectively). A clear separation between *O. citriodorum* essential oil and the four remaining *Ocimum* species was observed in the 3D score plot, indicating that the selected compounds were characteristic for sample discrimination (Figure 9(a)). Similarly, one hundred and fifty volatile selected compounds were subjected to HCA. The HCA is a powerful method to identify subgroups within a dataset, permitting observations with similar abundance profiles to merge into clusters. The result is displayed as a dendrogram (Figure 9(b)). *Ocimum* essential oil was classified into five clusters for the association of compounds detected in 5 different varieties of basil oil, with a cutoff = 0.05.

## 4. Discussion

The assessment of essential oil's chemical structure and composition in 5 basil varieties is of great significance due to their importance in different manufacturing. This study pioneers the use of a nontargeted approach with GC-MS to explore the existence of bioactive compounds in methanolic extracts of basil species [8, 9, 24–26]. This novel approach reveals a wealth of previously unexplored information that can influence the quality and utility of basil oils in various sectors. The GC-MS analysis, a basis of this study, uncovered a remarkable 111 distinct peaks in the essential oils of these basil species. Each peak represents a unique bioactive compound, and their identities were confirmed by matching their retention times and molecular formulas to known compounds as catalogued in the NIST library.

Comparing the GC data across five different *Ocimum* species, including *O. basilicum*, *O. gratissimum*, *O. sanctum*, *O. canum* and *O. citriodorum*, provides a captivating foretaste into the unique chemical profiles of their essential oils. *O. basilicum* distinguishes itself by its rich pathways of sesquiterpenoid and triterpenoid biosynthesis, then monoterpenoids and phenylpropanoid pathways, notably featuring the dominant compounds eugenol, estragole and caryophyllene, known for their pleasing, aromatic and anti-inflammatory properties, respectively [27–29]. The three compounds such as caryophyllene, *α*-humulene and caryophyllene belong to sesquiterpenoid and triterpenoid were found in all five *O. gratissimum* and *O. sanctum* contrast showed a distinct chemical signature with the presence of high Eugenol content. The eugenol is distinguished for its therapeutic properties, anti-inflammatory and antimicrobial effects [30–32]. Meanwhile, *O. canum* is chiefly defined by its monoterpene hydrocarbons, with linalool taking the lead. Linalool, renowned for its soothing and floral aroma, finds applications in the fragrance and cosmetics [33, 34]. *O. citriodorum* presents a chemical profile featuring sesquiterpenoid and triterpenoid biosynthesis, with caryophyllene as a standout compound known for its anti-inflammatory and antimicrobial properties [35, 36], rendering *O. sanctum* valuable in medicinal contexts [27, 37, 38]. These discrepancies in chemical composition among the *Ocimum* species underscore the diverse attributes and potential applications, underscoring the critical role of selecting the appropriate variety for specific purposes, whether culinary, therapeutic or aromatic.

These metabolic pathways confer distinct functions to various *Ocimum* species. *O. basilicum*, for instance, exhibits three dominant pathways alongside aminobenzoate degradation, tyrosine metabolism and toluene degradation. Aminobenzoate degradation is involved in breaking down aromatic compounds, contributing to nitrogen and carbon recycling for energy and essential molecules [39]. Tyrosine metabolism is crucial, converting tyrosine into secondary metabolites like phenolic compounds (flavonoids, coumarins and lignin), impacting plant defence, UV protection and structural support. It also contributes to the synthesis of melanin-like compounds and is linked to alkaloid biosynthesis for defensive functions [40]. *O. canum* specifically features the diterpenoid biosynthesis pathway, producing compounds that serve as defence mechanisms. *O. canum* and *O. sanctum* share pathways like xylene degradation and pinene, camphor and geraniol degradation, with *O. sanctum* having an additional valine, leucine and isoleucine degradation pathway [41]. *O. gratissimum* uniquely possesses the xylene degradation pathway [42]. While plants like Tulsi are recognized for their pharmacological properties, the microbial degradation of complex pollutants, such as xylene, is typically associated with specific soil bacteria. The presence of pinene, camphor and geraniol degradation pathways in *Ocimum* contributes to its unique characteristics by participating in the recycling of carbon and other elements [1–3].

The significant difference in the composition of essential oils in different *Ocimum* species was due to environmental factors, genetic differences and growth conditions. The comparative analysis of essential oil compositions in various *Ocimum* species reveals significant variations in the presence and abundance of chemical compounds. The compounds such as *α*-pinene, D-limonene and linalool are present in different quantities across the species, suggesting distinct aromatic profiles and therapeutic agents for malignant melanoma [3, 42, 43]. Additionally, the chemical composition of these essential oils is highly diverse, as seen from the varying percentages of compounds like estragole, *β*-caryophyllene, eugenol, linalool and camphor. Kholiya et al. described that *O. basilicum* (sweet basil) oil is often used for its calming and uplifting properties [27]. It is a common culinary herb and also has applications in aromatherapy. In a study conducted by [43], the antioxidant properties of essential oils derived from distinct varieties of *O. basilicum*, specifically *O. basilicum* var. purpureum and *O. basilicum* var. thyrsiflora, were explored. The findings from our research demonstrated that these particular varieties of basil are notably abundant in estragole, a compound recognized for its antioxidant attributes [4]. Consequently, these basil varieties exhibited the highest levels of antioxidant activity, underscoring their potential as a valuable source of natural antioxidants [4]. *O. basilicum* contains phenolic compounds such as 2-methoxy-4-butylphenol and diethyl phenol. According to a study by Bungau [44], these phenolic compounds exhibit potent antioxidant, antimicrobial, anti-inflammatory and antidiabetic properties. *O. sanctum* has key compounds, including eugenol, methyl chavicol (estragole), *β*-caryophyllene and camphor, and is valued for its adaptogenic and stress-relieving properties in traditional medicine [5, 24]. *O. gratissimum*, also known as African basil, has several important compounds like eugenol, *β*-caryophyllene, *α*-pinene and camphor for its antimicrobial and anti-inflammatory properties [5, 30, 31]. The major caryophyllene content ranged from 17.87% to 17.93% in *O. citriodorum* oil, which is higher than the literature reported earlier [19, 35]. This comprehensive chemical analysis provides valuable insights into the composition of essential oils in different basil varieties, with *O. citriodorum* standing out for its rich diversity of compounds, including caryophyllene as a major constituent. Caryophyllene has different valuable effects on nonalcoholic fatty liver disease/nonalcoholic steatohepatitis liver diseases, obesity, diabetes, cardiovascular diseases and other nervous system disorders [5]. *O. canum* rich in estragole, *α*-pinene, limonene and camphor has sweet and anise-like aroma. Due to *α*-pinene and limonene, *O. canum* essential oils have antioxidant and hypoglycaemic potential in diabetes mellitus, which may make it suitable for certain culinary dishes and potentially for aromatherapeutic purposes [7, 34]. *O. citriodorum*, due to the presence of compounds like methyl chavicol (estragole), *α*-pinene, *β*-caryophyllene and camphor, is used for its pleasant aroma and potential therapeutic benefits, similar to sweet basil [5, 45, 46]. *Ocimum* species are rich in terpenes, a diverse group of naturally occurring plant compounds that have emerged as potential therapeutic agents. They possess anticancer properties due to their ability to their ability to inhibit tumour initiation and induce apoptosis in tumour cells [3]. Additionally, these species exhibit antidiabetic, anti-inflammatory and antiallergic effects [4, 5]. They show promise in treating fatty liver disease/nonalcoholic, diabetes, cardiovascular diseases, pain and other nervous system disorders [5].

Indeed, these variations in chemical profiles among the *Ocimum* species play a pivotal role in shaping their essential oils' aroma, flavour and potential therapeutic properties. The unique chemical compositions influence the distinct scent and taste of the oils and determine their specific medicinal attributes. This understanding is paramount, particularly in industries like aromatherapy and herbal medicine, where selecting the right *Ocimum* species can be tailored to harness the desired chemical constituents for specific therapeutic or aromatic purposes. It underscores the significance of precise species selection based on their distinct chemical compositions to maximize the potential benefits in these specialized fields.

The PCA and HCA analyses provide valuable insights into the chemodiversity of *Ocimum* species, revealing distinct metabolic profiles that reflect species-specific biosynthetic pathways. The clear separation of *O. canum* and *O. basilicum* suggests the presence of unique metabolites, which could be linked to specialized ecological adaptations or functional properties. This detailed metabolic differentiation enhances our understanding of chemical variability within *Ocimum* and its potential applications. By identifying unique and shared metabolites across species, these analyses can guide future research on the genetic basis of metabolic traits and support breeding programmes to enhance essential oil composition for pharmaceutical, cosmetic and culinary uses. Such findings deepen our knowledge of basil chemodiversity and its relationship with genetic and environmental influences [46, 47].

## 5. Conclusion

The present study represents a significant advancement in essential oil analysis, specifically in the context of five different *Ocimum* species. By employing an untargeted approach with GC-MS, this research offers a detailed and comprehensive understanding of the diverse composition of essential oils in these species. The ability to clearly differentiate between *Ocimum* species and quantify the major pathways is sesquiterpenoid and triterpenoid biosynthesis compounds, such as D-limonene, linalool, caryophyllene, estragole, eugenol and more, is a pivotal achievement. Importantly, this study fills a notable gap in scientific literature, as there was no prior report of a comparative quantification study using GC-MS scan mode for these basil oils. This work not only enhances our understanding of the chemical profiles of basil oils but also holds practical implications for pharmaceutical and cosmetic industries, offering a basis for quality control and screening of raw materials. Identifying the varying chemical characteristics emphasizes the importance of precise botanical identification and knowledge of the plant's origin, particularly in traditional medicine applications. Ultimately, this research serves as a valuable resource for industries looking to harness the potential of these enriched molecules for perfumery and healthcare products.

Moreover, the use of statistical tools like PCA and HCA contributes to the depth of this study, enabling the visualization and distinction of these chemical profiles. The application of PCA highlights the clear separation between *O. citriodorum* and the other *Ocimum* species, underscoring the significance of the selected compounds in discerning between these varieties. The HCA results further enrich our understanding, providing insights into the associations between compounds and revealing resemblances among the five different *Ocimum* species. In a broader context, this work serves as a stepping stone for future research endeavours, offering opportunities to isolate and study specific bioactive compounds or optimize cultivation and extraction techniques for these basil oils. Overall, the findings from this study have the potential to transform the utilization of *Ocimum* essential oils across various industries, offering a rich source of industrially important molecules for developing products in perfumery, healthcare and beyond.

## Figures and Tables

**Table 1:** List of metabolites detected in different *Ocimum* spp.

S. no.	Name	Formula	RT	RI (experimental)	*Ocimum basilicum* (3)			*O. canum* (3)			*Ocimum citriodorum* (3)			*Ocimum sanctum* (3)			*Ocimum gratissimum* (3)			Identification method
					1	2	3	1	2	3	1	2	3	1	2	3	1	2	3	
Contents (%)																				
1	*α*-Pinene	C_10_H_16_	6.421	934	—	—	—	1.59	1.65	1.61	—	—	—	0.16	0.17	0.16	—	—	—	Wiley/NIST 2020 Mass Library; RI
2	Sulcatone	C_8_H_14_O	7.917	986	—	—	—	0.39	0.39	0.39	—	—	—	—	—	—	—	—	—	Wiley/NIST 2020 Mass Library; RI
3	*β*-Pinene	C_10_H_16_	8.058	991	—	—	—	0.28	0.29	0.32	0	0.19	0.2	—	—	—	—	—	—	Wiley/NIST 2020 Mass Library; RI
4	*p*-Cymene	C_10_H_14_	9.225	1025	—	—	—	0.19	0.19	0.2	—	—	—	0.36	0.36	0.31	1.42	1.45	1.57	Wiley/NIST 2020 Mass Library; RI
5	D-limonene	C_10_H_16_	9.376	1029	—	—	—	3.05	3.06	3.3	0.64	0.61	0.64	0.18	0.18	0.17	—	—	—	Wiley/NIST 2020 Mass Library; RI
6	Eucalyptol	C_10_H_18_O	9.473	1031	0.20	0.20	0.53	0.56	0.56	0.56	—	—	—	1.01	1.05	1.02	—	—	—	Wiley/NIST 2020 Mass Library; RI
7	*β*-Ocimene	C_10_H_16_	10.056	1047	—	—	—	—	—	—	0.18	0.18	0.2	—	—	—	—	—	—	Wiley/NIST 2020 Mass Library; RI
8	(E)-Linalool oxide A	C_10_H_18_O_2_	11.007	1073	0.95	3.79	3.73	2.06	2.02	1.94	—	—	—	—	—	—	—	—	—	Wiley/NIST 2020 Mass Library; RI
9	2-Carene	C_10_H_16_	11.623	1089	—	—	—	—	—	—	0.31	0.29	0.3	—	—	—	—	—	—	Wiley/NIST 2020 Mass Library; RI
10	cis-Linaloloxide	C_10_H_18_O_2_	11.628	1089	0.95	3.66	3.60	2.73	2.7	2.62	—	—	—	—	—	—	—	—	—	Wiley/NIST 2020 Mass Library; RI
11	Linalool	C_10_H_18_O	12.076	1101	14.30	13.96	20.33	51.26	50.99	50.81	3.62	3.66	3.57	—	—	—	3.28	3.33	3.33	Wiley/NIST 2020 Mass Library; RI
12	E-myrtenol	C_10_H_18_O	12.612	1114	—	—	—	0.27	0.26	0.25	—	—	—	—	—	—	—	—	—	Wiley/NIST 2020 Mass Library; RI
13	Linalool, methyl ether	C_11_H_20_O	13.465	1135	—	—	—	—	—	—	—	—	—	0.2	0.21	0.21	—	—	—	Wiley/NIST 2020 Mass Library; RI
14	Menthyl isovalerate	C_15_H_28_O_2_	13.6	1138	—	—	—	—	—	—	—	—	—	0.27	0.28	0.29	—	—	—	Wiley/NIST 2020 Mass Library; RI
15	1-Methoxy-3,5-dimethyl-cyclohexene	C_9_H_16_O	13.676	1140	—	—	—	0.36	0.37	0.38	—	—	—	—	—	—	—	—	—	Wiley/NIST 2020 Mass Library; RI
16	2-Bornanone	C_10_H_16_O	13.935	1146	—	—	—	2.10	2.11	2.08	—	—	—	—	—	—	3.87	3.88	3.89	Wiley/NIST 2020 Mass Library; RI
17	Borneol	C_10_H_18_O	14.4	1157	—	—	—	—	—	—	—	—	—	0.45	0.48	0.48	—	—	—	Wiley/NIST 2020 Mass Library; RI
18	Exo-norbornyl propionate	C_10_H_16_O_2_	14.924	1169	0.32	0.31	0.00	—	—	—	—	—	—	—	—	—	—	—	—	Wiley/NIST 2020 Mass Library; RI
19	(+)-Neoisomenthol	C_10_H_20_O	15.075	1173	0.00	0.00	0.27	—	—	—	—	—	—	—	—	—	—	—	—	Wiley/NIST 2020 Mass Library; RI
20	D-neoisomenthol	C_10_H_20_O	15.118	1174	0.53	0.52	0.00	—	—	—	—	—	—	—	—	—	—	—	—	Wiley/NIST 2020 Mass Library; RI
21	cis-3-Hexenyl butyrate	C_10_H_18_O_2_	15.783	1190	—	—	—	1.12	1.07	1.01	—	—	—	—	—	—	—	—	—	Wiley/NIST 2020 Mass Library; RI
22	*α*-Terpineol	C_10_H_18_O	15.826	1191	—	—	—	—	—	—	0.38	0.39	0.37	—	—	—	1.18	1.19	1.19	Wiley/NIST 2020 Mass Library; RI
23	cis-Ocimenol	C_10_H_16_O	15.853	1192	—	—	—	1.86	1.88	1.83	—	—	—	—	—	—	—	—	—	Wiley/NIST 2020 Mass Library; RI
24	Linalool-7-OH	C_10_H_18_O_2_	15.983	1195	1.03	1.06	0.34	—	—	—	—	—	—	—	—	—	—	—	—	Wiley/NIST 2020 Mass Library; RI
25	*β*-Terpinyl acetate	C_12_H_22_O_2_	16.133	1198	—	—	—	—	—	—	—	—	—	—	—	—	0.29	0.29	0.3	Wiley/NIST 2020 Mass Library; RI
26	Estragole	C_10_H_12_O	16.436	1206	49.49	48.55	61.05	0.75	0.79	0.78	—	—	—	0.43	0.46	0.46	—	—	—	Wiley/NIST 2020 Mass Library; RI
27	Citronellol	C_10_H_20_O	17.403	1228	—	—	—	—	—	—	—	—	—	0.88	0.98	0.97	—	—	—	Wiley/NIST 2020 Mass Library; RI
28	Anisaldehyde	C_8_H_8_O_2_	18.522	1254	0.65	0.62	0.00	—	—	—	—	—	—	—	—	—	—	—	—	Wiley/NIST 2020 Mass Library; RI
29	Geraniol	C_10_H_18_O	18.532	1254	—	—	—	—	—	—	0.24	0.25	0.24	—	—	—	—	—	—	Wiley/NIST 2020 Mass Library; RI
30	(E)-Cinnamaldehyde	C_9_H_8_O	19.207	1270	—	—	—	—	—	—	0.4	1.19	1.62	—	—	—	—	—	—	Wiley/NIST 2020 Mass Library; RI
31	D-neoisomenthol	C_10_H_20_O	19.326	1273	0.46	0.46	0.00	0.22	0.18	0.2	—	—	—	—	—	—	—	—	—	Wiley/NIST 2020 Mass Library; RI
32	Decahydronaphthalen-2-ol	C_10_H_18_O	20.002	1289	—	—	—	2.50	2.48	2.48	—	—	—	—	—	—	—	—	—	Wiley/NIST 2020 Mass Library; RI
33	Nerolidol-epoxy acetate	C_17_H_28_O_4_	20.482	1300	0.15	0.18	0.00	—	—	—	—	—	—	—	—	—	—	—	—	Wiley/NIST 2020 Mass Library; RI
34	Neryl acetal	C_12_H_22_O_2_	21.260	1318	0.35	0.35	0.91	—	—	—	—	—	—	—	—	—	—	—	—	Wiley/NIST 2020 Mass Library; RI
35	Geranyl acetal	C_12_H_22_O_2_	22.233	1341	0.73	0.75	1.73	—	—	—	—	—	—	—	—	—	—	—	—	Wiley/NIST 2020 Mass Library; RI
36	*α*-Cubebene	C_15_H_24_	22.649	1351	—	—	—	—	—	—	—	—	—	0.23	0.22	0.22	0.18	0.18	0.2	Wiley/NIST 2020 Mass Library; RI
37	2,3,4,6-Tetramethylphenol	C_10_H_14_O	22.714	1353	0.00	0.16	0.00	—	—	—	—	—	—	—	—	—	—	—	—	Wiley/NIST 2020 Mass Library; RI
38	Nerolidol Z and E	C_15_H_26_O	22.984	1359	0.30	0.33	0.00	—	—	—	—	—	—	—	—	—	—	—	—	Wiley/NIST 2020 Mass Library; RI
39	Eugenol	C_10_H_12_O_2_	23.032	1360	—	—	—	1.15	1.29	1.39	11.5	11.49	11.61	52.6	52.14	52.21	58.67	59.29	59.83	Wiley/NIST 2020 Mass Library; RI
40	Isocaucalol	C_15_H_26_O_3_	23.184	1364	0.00	0.16	0.00	—	—	—	—	—	—	—	—	—	—	—	—	Wiley/NIST 2020 Mass Library; RI
41	*α*-Guaiene	C_15_H_24_	23.307	1367	—	—	—	—	—	—	0.54	0.56	0.52	—	—	—	—	—	—	Wiley/NIST 2020 Mass Library; RI
42	Anisaldehyde dimethyl acetal	C_10_H_14_O_3_	23.356	1368	0.32	2.85	2.91	—	—	—	—	—	—	—	—	—	—	—	—	Wiley/NIST 2020 Mass Library; RI
43	Longifolene-(V4)	C_15_H_24_	23.513	1372	—	—	—	—	—	—	0.52	0.52	0.5	—	—	—	—	—	—	Wiley/NIST 2020 Mass Library; RI
44	Ylangene	C_15_H_24_	23.588	1373	—	—	—	—	—	—	0.26	0.23	0.21	—	—	—	—	—	—	Wiley/NIST 2020 Mass Library; RI
45	Copaene	C_15_H_24_	23.756	1377	—	—	—	0.23	0.23	0.22	1.67	1.63	1.59	0.89	0.93	0.9	0.88	0.85	0.8	Wiley/NIST 2020 Mass Library; RI
46	Isomyrcenyl acetate	C_12_H_18_O_2_	24.161	1387	0.20	0.22	0.00	—	—	—	—	—	—	—	—	—	—	—	—	Wiley/NIST 2020 Mass Library; RI
47	*β*-Selinene	C_15_H_24_	24.453	1394	—	—	—	—	—	—	0	0.21	0	—	—	—	—	—	—	Wiley/NIST 2020 Mass Library; RI
48	*β*-Clovene	C_15_H_24_	24.566	1397	—	—	—	—	—	—	0	0.2	0	—	—	—	—	—	—	Wiley/NIST 2020 Mass Library; RI
49	Cinnamaldehyde dimethyl acetal	C_11_H_14_O_2_	24.755	1401	—	—	—	—	—	—	2.38	3.09	4.17	—	—	—	—	—	—	Wiley/NIST 2020 Mass Library; RI
50	Methyleugenol	C_11_H_14_O_2_	24.934	1405	0.16	0.18	0.00	2.94	2.92	2.92	—	—	—	—	—	—	—	—	—	Wiley/NIST 2020 Mass Library; RI
51	*α*-Elemene	C_15_H_24_	25.09	1409	—	—	—	—	—	—	2.75	2.74	2.55	0.17	0.16	0.16	—	—	—	Wiley/NIST 2020 Mass Library; RI
52	*α*-Neoclovene	C_15_H_24_	25.322	1415	—	—	—	—	—	—	3.79	3.52	3.77	—	—	—	—	—	—	Wiley/NIST 2020 Mass Library; RI
53	Caryophyllene	C_15_H_24_	25.496	1421	0.40	0.43	0.40	7.66	7.84	7.98	17.93	17.98	17.87	26.16	25.83	25.43	17.69	17.89	18.61	Wiley/NIST 2020 Mass Library; RI
54	*β*-Gurjunene	C_15_H_24_	25.96	1431	—	—	—	—	—	—	0.53	0.53	0.52	0.18	0.19	0.21				Wiley/NIST 2020 Mass Library; RI
55	*γ*-Cadinene	C_15_H_24_	26.079	1434	—	—	—	—	—	—	1.23	1.27	1.25	—	—	—				Wiley/NIST 2020 Mass Library; RI
56	*α*-Longipinene	C_15_H_24_	26.219	1437	1.26	1.29	1.83				1.06	1.07	1.07	—	—	—	0.75	0.74	0.74	Wiley/NIST 2020 Mass Library; RI
57	*α*-Cadinene	C_15_H_24_	26.36	1441	—	—	—	—	—	—	0.21	0.21	0.21	—	—	—	—	—	—	Wiley/NIST 2020 Mass Library; RI
58	*β*-Panasinsene	C_15_H_24_	26.478	1443	—	—	—	—	—	—	0.28	0.3	0.29	—	—	—	—	—	—	Wiley/NIST 2020 Mass Library; RI
59	Cedrene-V6	C_15_H_24_	26.592	1446	—	—	—	0.60	0.59	0.6	—	—	—	—	—	—	—	—	—	Wiley/NIST 2020 Mass Library; RI
60	Isocaryophyllene	C_15_H_24_	26.797	1451	—	—	—	0.24	0.24	0.24	—	—	—	—	—	—	—	—	—	Wiley/NIST 2020 Mass Library; RI
61	Aromadendrene	C_15_H_24_	26.889	1454	—	—	—	—	—	—	7.11	7.22	7.17	—	—	—	—	—	—	Wiley/NIST 2020 Mass Library; RI
62	*α*-Humulene	C_15_H_24_	26.954	1455	0.00	0.00	0.26	3.71	3.77	3.8	6.02	6.1	6.05	6.93	6.97	6.91	4.25	4.28	4.41	Wiley/NIST 2020 Mass Library; RI
63	*α*-Bisabolene	C_15_H_24_	27.051	1458	—	—	—	—	—	—	—	—	—	—	—	—	—	—	—	Wiley/NIST 2020 Mass Library; RI
64	Longifolene	C_15_H_24_	27.246	1462	—	—	—	—	—	—	0.21	0.2	0.2	—	—	—	—	—	—	Wiley/NIST 2020 Mass Library; RI
65	Valencene	C_15_H_24_	27.775	1475	—	—	—	—	—	—	—	—	—	0.17	27.781	27.786	—	—	—	Wiley/NIST 2020 Mass Library; RI
66	*γ*-Cadinene	C_15_H_24_	27.889	1478	—	—	—	—	—	—	0.25	0.25	0.24	0.16	0.17	0.17	—	—	—	Wiley/NIST 2020 Mass Library; RI
67	*δ*-Selinene	C_15_H_24_	28.029	1482	—	—	—	—	—	—	0.19	0.19	0.19	—	—	—	—	—	—	Wiley/NIST 2020 Mass Library; RI
68	*α*-Curcumene	C_15_H_22_	28.132	1484	—	—	—	0.16	0.16	0.16	—	—	—	—	—	—	—	—	—	Wiley/NIST 2020 Mass Library; RI
69	Germacrene D	C_15_H_24_	28.207	1486	—	—	—	0.20	0.21	0.28	—	—	—	—	—	—	—	—	—	Wiley/NIST 2020 Mass Library; RI
70	*α*-Selinene	C_15_H_24_	28.634	1497	—	—	—	—	—	—	0.28	0.28	0.28	0.21	0.21	0.22				Wiley/NIST 2020 Mass Library; RI
71	Neoalloocimene	C_15_H_24_	28.634	1497	—	—	—	—	—	—	—	—	—	—	—	—	—	—	—	Wiley/NIST 2020 Mass Library; RI
72	*γ*-Cadinene	C_15_H_24_	28.737	1499				0.82	0.82	0.82	—	—	—	—	—	—	—	—	—	Wiley/NIST 2020 Mass Library; RI
73	*γ*-Muurolene	C_15_H_24_	28.78	1500	—	—	—	—	—	—	0.45	0.46	0.47	—	—	—	—	—	—	Wiley/NIST 2020 Mass Library; RI
74	*γ*-Selinene	C_15_H_24_	28.839	1502	—	—	—	0.18	0.19	0.19	—	—	—	—	—	—	—	—	—	Wiley/NIST 2020 Mass Library; RI
75	*α*-Muurolene	C_15_H_24_	28.839	1502	—	—	—	—	—	—	0.36	0.37	0.36	0.17	0.17	0.18				Wiley/NIST 2020 Mass Library; RI
76	*α*-Himachalene	C_15_H_24_	29.158	1510	0.16	0.17	0.26	0.96	0.96	0.96	0.33	0.35	0.35	—	—	—	—	—	—	Wiley/NIST 2020 Mass Library; RI
77	D-germacrene	C_15_H_24_	29.380	1516	—	—	—	0.41	0.41	0.43	—	—	—	—	—	—	—	—	—	Wiley/NIST 2020 Mass Library; RI
78	*β*-Spathulenol	C_15_H_24_O	29.423	1517	—	—	—	—	—	—	0.7	0.7	0.71	—	—	—	—	—	—	Wiley/NIST 2020 Mass Library; RI
79	*δ*-Cadinene	C_15_H_24_	29.752	1525	—	—	—	0.68	0.71	0.71	2.01	1.99	2	1.43	1.48	1.5	0.95	0.95	0.96	Wiley/NIST 2020 Mass Library; RI
80	*α*-Cubebene	C_15_H_24_	30.098	1534	—	—	—	—	—	—	0.2	0.2	0.2	—	—	—	—	—	—	Wiley/NIST 2020 Mass Library; RI
81	*α*-Bisabolene	C_15_H_24_	30.492	1544	0.21	0.22	2.75	4.53	4.89	5.22	0.78	0.76	0.77	—	—	—	0.3	0.41	0.33	Wiley/NIST 2020 Mass Library; RI
82	Guaiol	C_15_H_26_O	30.741	1551	—	—	—	—	—	—	—	—	—	0.17	0.19	0.2				Wiley/NIST 2020 Mass Library; RI
83	Betulenol	C_15_H_24_O	30.865	1554	—	—	—	—	—	—	—	—	—	0.29	0.28	0.25	0.35	0.37	0.35	Wiley/NIST 2020 Mass Library; RI
84	Humulene epoxide I	C_15_H_24_O	30.892	1555	—	—	—	—	—	—	0.25	0.24	0.25	—	—	—	—	—	—	Wiley/NIST 2020 Mass Library; RI
85	Bicyclogermacrene	C_15_H_24_	31.033	1558	—	—	—	0.57	0.55	0.55				—	—	—	—	—	—	Wiley/NIST 2020 Mass Library; RI
86	Longifolenaldehyde	C_15_H_24_O	31.14	1561	—	—	—	0.2	0.19	0.2	0.19	0.19	0.19	—	—	—	—	—	—	Wiley/NIST 2020 Mass Library; RI
87	4-Methoxycinnamaldehyde	C_10_H_10_O_2_	31.432	1568	2.77	5.20	5.19	—	—	—				—	—	—	—	—	—	Wiley/NIST 2020 Mass Library; RI
88	Caryophyllenyl alcohol	C_15_H_26_O	31.6	1573	—	—	—	—	—	—	4.78	4.83	4.82	—	—	—	—	—	—	Wiley/NIST 2020 Mass Library; RI
89	Isospathulenol	C_15_H_24_O	31.854	1579	0.00	0.19	0.00	—	—	—				—	—	—	—	—	—	Wiley/NIST 2020 Mass Library; RI
90	Fonenol	C_15_H_26_O	31.913	1581	—	—	—	—	—	—	2.47	2.37	2.61	—	—	—	—	—	—	Wiley/NIST 2020 Mass Library; RI
91	Caryophyllene oxide	C_15_H_24_O	32.070	1585	1.14	1.01	1.27	1.75	1.48	1.31	0.39	32.075	32.075	4.16	4.22	4.58	2.38	3.57	3.36	Wiley/NIST 2020 Mass Library; RI
92	Globulol	C_15_H_26_O	32.075	1585	—	—	—	—	—	—				—	—	—	—	—	—	Wiley/NIST 2020 Mass Library; RI
93	*α*-Elemene	C_15_H_24_	32.14	1587	1.29	1.33	0.00	—	—	—	0.28	0.28	0.25	—	—	—	—	—	—	Wiley/NIST 2020 Mass Library; RI
94	2-Methoxybenzyl acetate	C_10_H_12_O_3_	32.156	1587	—	—	—	—	—	—				—	—	—	—	—	—	Wiley/NIST 2020 Mass Library; RI
95	2-Methoxy-4-butylphenol	C_11_H_16_O_2_	32.221	1589	1.20	1.27	0.00	—	—	—				—	—	—	—	—	—	Wiley/NIST 2020 Mass Library; RI
96	Neoalloocimene	C_15_H_24_	32.453	1595	—	—	—	—	—	—	0.18	0.19	0.18	—	—	—	—	—	—	Wiley/NIST 2020 Mass Library; RI
97	Ledol	C_15_H_26_O	32.766	1603	—	—	—	—	—	—	1.33	1.31	1.36	—	—	—	—	—	—	Wiley/NIST 2020 Mass Library; RI
98	Diisobutyl	C_11_H_25_O_3_P	32.95	1608	—	—	—	—	—	—	0	0	0.22	—	—	—	—	—	—	Wiley/NIST 2020 Mass Library; RI
99	Humulene oxide II	C_15_H_24_O	33.058	1611	0.60	0.51	0.65	0.45	0.37	0.34				0.6	0.64	0.72	0.46	0.42	0.27	Wiley/NIST 2020 Mass Library; RI
100	Widdrol	C_15_H_26_O	33.366	1619	—	—	—	—	—	—	0.6	0.59	0.54	—	—	—	—	—	—	Wiley/NIST 2020 Mass Library; RI
101	*p*-Methoxy-α-methyl-cinnamic acid	C_11_H_12_O_3_	33.436	1621	0.59	0.59	0.00	—	—	—				—	—	—	—	—	—	Wiley/NIST 2020 Mass Library; RI
102	Nepetalactone	C_10_H_14_O_2_	33.831	1632	—	—	—	—	—	—	1.12	1.12	1.15	—	—	—	—	—	—	Wiley/NIST 2020 Mass Library; RI
103	Isolongifolol	C_15_H_26_O	33.912	1634	—	—	—	—	—	—	0.3	0.36	0.42	—	—	—	—	—	—	Wiley/NIST 2020 Mass Library; RI
104	*γ*-Eudesmol	C_13_H_11_NO_3_	34.052	1638	—	—	—	—	—	—				0.18	0.21	0.23	0.19	0.11	0.15	Wiley/NIST 2020 Mass Library; RI
105	Duvatriendiol	C_20_H_34_O_2_	34.382	1647	0.26	0.27	0.00	—	—	—				—	—	—	—	—	—	Wiley/NIST 2020 Mass Library; RI
106	Nerolidol-epoxy acetate	C_17_H_28_O_4_	34.517	1650	0.19	0.20	0.00	—	—	—	0.3	0.34	0.32	0.17	0.2	0.22	0.21	0.2	0.18	Wiley/NIST 2020 Mass Library; RI
107	Phenol, diethyl-	C_10_H_14_O	34.949	1662	0.21	0.23	0.00	—	—	—				—	—	—	—	—	—	Wiley/NIST 2020 Mass Library; RI
108	1,2-Dimethoxy-4-(3-propenyl)benzene	C_12_H_16_O_3_	35.316	1672	2.20	4.20	4.34	—	—	—				—	—	—	—	—	—	Wiley/NIST 2020 Mass Library; RI
109	Solanesol	C_45_H_74_O	35.327	1672	—	—	—	—	—	—	0.23	0.25	0.26	—	—	—	—	—	—	Wiley/NIST 2020 Mass Library; RI
110	*α*-Cyperone	C_15_H_21_DO	35.333	1672	—	—	—	0.25	0.21	0.23				0.76	0.92	0.69	0.34	0.64	0.56	Wiley/NIST 2020 Mass Library; RI
111	Neoalloocimene	C_15_H_24_	65.73	2743	—	—	—	—	—	—	—	—	—	—	—	—	0.18	0.00	0.00	Wiley/NIST 2020 Mass Library; RI

*Note:* Compounds were identified by comparison of their experimental retention indices (RI) and mass spectra with those of authentic compounds on the HP-5MS UI column compounds on the HP-5MS UI column. The % content is the % peak area of the total essential oil composition.

## Data Availability

The data that support the findings of this study are available from the corresponding author upon reasonable request.

## Nomenclature

GC-MS: Gas chromatography–mass spectrometry
LC-MS: Liquid chromatography–mass spectrometry
KEGG: Kyoto Encyclopedia of Genes and Genomes
NBPGR: National Bureau of Plant Genetic Resources
PCA: Principle component analysis
HCA: Hierarchical clustering analysis
NIST: National Institute of Standards and Technology
RI: Retention indices
UV: Ultraviolet

## Supporting Information

The list of specific metabolites identified in different species (*Ocimum basilicum*, *O. canum*, *O. citriodorum*, *O. sanctum* and *O. gratissimum*) along with their retention times (RT), indicating their elution order in chromatographic analysis, is provided as Supporting data in Tables S1 to S5. Table S1: List of specific metabolites detected in *Ocimum basilicum* by GC-MS analysis. Table S2: List of specific metabolites detected in *Ocimum canum* by GC-MS analysis. Table S3: List of specific metabolites detected in *Ocimum citriodorum* by GC-MS analysis. Table S4: List of specific metabolites detected in *Ocimum sanctum* by GC-MS analysis. Table S5: List of specific metabolites detected in *Ocimum gratissimum* by GC-MS analysis.
