# Supplementary material for: Screening of Metabolites and Metabolic Pathways in Five Different Ocimum Species From the Same Origin Using GC-MS
Source: Biochem Res Int. 2025 Jun 24;2025:7121687. doi: 10.1155/bri/7121687 (PMC12213051; doi:10.1155/bri/7121687)
Supplement: Supporting Information — Additional supporting information can be found online in the Supporting Information section. [file 7121687.f1.docx]

**Screening of metabolites and metabolic pathways in five different *Ocimum* species from the same origin using GC–MS**

**Ravi Prakash Jaiswal^1^, Vishal Chugh^2^, Sushil Nagar^3^, Shalini Purwar^4^*, Akbare Azam^1^, Ankit Verma^2,4^**

^1^Department of Chemistry, ^1^Government Girls P.G. College, Ghazipur (Affiliated to Veer Bahadur Singh Purvanchal University), Jaunpur, Uttar Pradesh - 233001, India.

^2^Department of Basic and Social Sciences, College of Horticulture, Banda University of Agriculture and Technology, Banda, Uttar Pradesh-210001, India.

^3^Department of Biochemistry, College of Basic Sciences and Humanities, CCS Haryana Agricultural University, Hisar, Haryana, 125004, India

^4^Department of Basic and Social Sciences, College of forestry, Banda University of Agriculture and Technology, Banda, Uttar Pradesh-210001, India.

**Correspondence Author:** [purwarshalini@gmail.com](mailto:purwarshalini@gmail.com), [shalinipurwar@buat.edu.in](mailto:shalinipurwar@buat.edu.in)

**Supplementary Tables**

**Table S1 : List of specific metabolites identified in Ocimum basilicum along with their retention times (RT), indicating their elution order in chromatographic analysis.**

| **S. No.** | ***O. Basilicum*** | **RT** |
| --- | --- | --- |
| 1. | exo-Norbornyl propionate | 0.32 |
| 2. | (+)-Neoisomenthol | 0.27 |
| 3. | D-neoisomenthol | 0.53 |
| 4. | Linalool-7-OH | 0.81 |
| 5. | Anisaldehyde | 0.42 |
| 6. | Nerolidol-epoxyacetate | 0.11 |
| 7. | Nerylacetal | 0.54 |
| 8. | Geranylacetal | 1.07 |
| 9. | 2,3,4,6-Tetramethylphenol | 0.05 |
| 10. | Nerolidol Z and E | 0.21 |
| 11. | Isocaucalol | 0.05 |
| 12. | Anisaldehyde dimethyl acetal | 2.03 |
| 13. | Isomyrcenylacetat | 0.14 |
| 14. | 4-Methoxycinnamaldehyde | 4.39 |
| 15. | Isospathulenol | 0.06 |
| 16. | α-Elemene | 0.87 |
| 17. | 2-Methoxy-4-butylphenol | 0.82 |
| 18. | p-Methoxy-α-methyl-cinnamic acid | 0.39 |
| 19. | Duvatriendiol | 0.18 |
| 20. | Phenol, diethyl- | 0.13 |
| 21. | 1,2-Dimethoxy-4-(3- | 0.15 |
| 22. | 1,2-Dimethoxy-4-(3- | 3.58 |

**Table S2: List of specific metabolites identified in *O. canum* along with their retention times (RT), indicating their elution order in chromatographic analysis.**

| **S. No.** | ***O. Canum*** | **RT** |
| --- | --- | --- |
|  | Sulcatone | 0.39 |
|  | E-myrtenol | 0.26 |
|  | 1-Methoxy-3,5-dimethyl-cyclohexene | 0.37 |
|  | cis-3-Hexenyl butyrate | 1.07 |
|  | cis-Ocimenol | 1.86 |
|  | Decahydronaphthalen-2-ol | 2.49 |
|  | Cedrene-V6 | 0.60 |
|  | Isocaryophyllene | 0.24 |
|  | α-Curcumene | 0.46 |
|  | Germacrene D | 0.15 |
|  | γ-Cadinene | 0.82 |
|  | γ-Selinene | 0.19 |
|  | D-Germacrene | 0.42 |
|  | Bicyclogermacrene | 0.56 |

**Table S3: List of specific metabolites identified in *O. citriodorum* along with their retention times (RT), indicating their elution order in chromatographic analysis.**

| **S. No.** | ***O. Citriodorum*** | **RT** |
| --- | --- | --- |
|  | β-Ocimene | 0.19 |
|  | 2-Carene | 0.30 |
|  | α-Terpineol | 0.38 |
|  | Geraniol | 0.24 |
|  | (E)-Cinnamaldehyde | 1.07 |
|  | α-Guaiene | 0.54 |
|  | Longifolene-(V4) | 0.51 |
|  | Y langene | 0.23 |
|  | β-Selinene | 0.07 |
|  | β-Clovene | 0.07 |
|  | Cinnamaldehyde dimethyl acetal | 3.21 |
|  | α-Neoclovene | 3.69 |
|  | γ-Cadinene | 1.25 |
|  | α-Cadinene | 0.21 |
|  | β-Panasinsene | 0.29 |
|  | Aromandendrene | 7.17 |
|  | Longifolene | 0.20 |
|  | δ-Selinene | 0.19 |
|  | γ-Muurolene | 0.46 |
|  | β-Spathulenol | 0.70 |
|  | α-Cubebene | 0.20 |
|  | Humulene epoxide I | 0.25 |
|  | Caryophyllenyl alcohol | 4.81 |
|  | Fonenol | 2.48 |
|  | α-Elemene | 0.27 |
|  | Neoalloocimene | 0.18 |
|  | Ledol | 1.33 |
|  | Diisobutyl | 0.07 |
|  | Widdrol | 0.58 |
|  | Nepetalactone | 1.13 |
|  | Isolongifolol | 0.36 |
|  | Solanesol | 0.25 |

**Table S4: List of specific metabolites identified in *O. sanctum* along with their retention times (RT), indicating their elution order in chromatographic analysis.**

| **S. No.** | ***O. Sanctum*** | **RT** |
| --- | --- | --- |
|  | Linalool, methyl ether | 0.21 |
|  | Menthylisovalerate | 0.28 |
|  | Borneol | 0.47 |
|  | Citronellol | 0.94 |
|  | Valencene | 18.58 |
|  | Guaiol | 0.19 |

**Table S5: The specific metabolites of*.* List of specific metabolites identified in *O. gratissimum* along with their retention times (RT), indicating their elution order in chromatographic analysis.**

| **S. No.** | ***O. Gratissimum*** | **RT** |
| --- | --- | --- |
|  | Neoalloocimene | 0.18 |
|  | β-Terpinyl acetate | 0.29 |
